# The emerging role of circular RNAs in Parkinson’s disease

**DOI:** 10.3389/fnins.2023.1137363

**Published:** 2023-02-16

**Authors:** Jiajia Liao, Qinxin Zhang, Jinjun Huang, Honghu He, Jiang Lei, Yuefei Shen, Jin Wang, Yousheng Xiao

**Affiliations:** ^1^Department of Neurology, The First Affiliated Hospital of Guangxi Medical University, Nanning, China; ^2^Department of Rehabilitation Medicine, Jiangbin Hospital of Guangxi Zhuang Autonomous Region, Nanning, China; ^3^Department of Rehabilitation, Guiping People’s Hospital, Guiping, China

**Keywords:** circRNAs, conservation, stability, biogenesis, miRNA sponge, translation, Parkinson’s disease

## Abstract

Parkinson’s disease (PD) is the second most prevalent neurodegenerative disease and the most common movement disorder. It involves a gradual loss of dopaminergic neurons in the substantia nigra. Although many studies have been conducted, the underlying molecular pathways of PD remain largely unknown. Circular RNAs (circRNAs), a novel class of non-coding RNAs with a covalently closed loop structure, are common in the brain. They are stable, conserved molecules that are widely expressed in eukaryotes in tissue-, cell-, and development-specific patterns. Many circRNAs have recently been identified in nervous system diseases, and some circRNA expression profiles have been linked to PD. Given that recent research has indicated the essential roles of various circRNAs in the development and progression of neurodegenerative diseases, the identification of individual circRNAs may be a promising strategy for finding new treatment targets for PD. Moreover, the search for circRNAs with high specificity and sensitivity will open up new avenues for the early diagnosis and treatment of PD. Herein, we address the biogenesis, properties, and roles of circRNAs and review their potential utility as biomarkers and therapeutic targets in PD.

## Introduction

Parkinson’s disease (PD) is the second most common neurodegenerative disease and affects millions of middle-aged and older people worldwide. Motor symptoms such as bradykinesia, resting tremor, postural abnormalities, and muscular rigidity as well as non-motor symptoms such as sleep disturbance, hallucinations, and autonomic nervous dysfunction are the most common clinical symptoms of PD. Phenotypic similarities to other atypical parkinsonian disorders, such as multisystem atrophy or corticobasal degeneration, complicate the diagnosis of PD; the symptoms of these disorders overlap with those of PD, particularly in its early stages. But the pathological differences between PD and other parkinsonism syndromes are that PD is characterized by the large-scale loss of dopaminergic (DA) neurons in the substantia nigra (SN) pars compacta as well as a gradual accumulation of intracellular alpha-synuclein (SNCA or α-syn). Epidemiological research indicates that the prevalence rate of PD in individuals over 65 years of age in China is around 1.7% (Lin et al., 2021). Moreover, the incidence rate of PD is rising, leading to an important burden on patients’ families and society. PD is mainly diagnosed based on clinical symptoms and can be assessed using a variety of rating scales for motor and non-motor symptoms (Postuma et al., 2015). Such rating scales are often subjective and can be influenced by changes in symptoms or effective treatments. Furthermore, although dopamine transporter single-photon emission computed tomography can be used to show how quickly PD is progressing, its practicality and cost often hinder its use (Bhidayasiri and Martinez-Martin, 2017). Additionally, biomarkers based on α-syn and the metabolic products of dopamine have generated conflicting outcomes (Miller and O’Callaghan, 2015). Despite efforts to investigate the pathogenesis of PD, its exact etiology remains largely unknown, and specific and meaningful biomarkers for its diagnosis remain limited. As a result, the identification of new biomarkers and therapeutic targets remains an important challenge.

Noncoding RNAs (ncRNAs) are involved in a range of cellular biological and physiological activities as well as in many pathological disease processes (Goodall and Wickramasinghe, 2021). Circular RNAs (circRNAs), which are generated via the back-splicing of precursor messenger RNAs (mRNAs) or other specific RNA molecules (Chen, 2020), have high stability, abundance, and conservation, and display tissue- or developmental stage-specific expression (Zlotorynski, 2019). They have thus been the focus of intense research in recent years. CircRNAs were first thought to be spliced intermediates or by-products of erroneous splicing (Kristensen et al., 2019). As high-throughput sequencing techniques have advanced, circRNAs have been demonstrated to control gene expression by influencing the transcription of target genes and splicing, functioning as microRNA (miRNA or miR) sponges, interacting with proteins, and converting their own RNA sequences into peptides (Lei et al., 2020; Okholm et al., 2020).

Accumulating evidence indicates that both protein-coding and ncRNAs play a role in the development of PD. Long ncRNAs and miRNAs are the two most prevalent linear ncRNA transcripts and have been identified in dozens of studies. By contrast, circRNA profiling has been performed much less frequently. However, recent research has revealed that numerous circRNAs are abnormally expressed in PD; these biochemically active molecules are thought to have an important role in the development of the disease and are being investigated as potential disease markers or drug targets (Sang et al., 2018; Feng et al., 2020; Hanan et al., 2020; Wang et al., 2021b). Nevertheless, the fundamental process and clinical significance of circRNAs in PD remain unclear. We will therefore detail the characteristics, biogenesis, mechanisms, and functions of circRNAs in this review. We will also address the significance of aberrantly generated circRNAs in PD and discuss the potential value of circRNAs as clinical markers and/or therapeutic targets in this disease.

## Biogenesis and classification of circRNAs

The exact mechanism of circRNA biosynthesis is yet to be discovered. CircRNAs are predominantly produced from precursor mRNAs through a type of alternative splicing known as back-splicing, according to a well-accepted method. A downstream 5′ splice site (the splice donor site) is linked to an upstream 3′ splice site (the splice acceptor site) in the reverse order during back-splicing reactions (Chen, 2020), which can be assisted by reverse complementary Alu repeats bordering the circularized exon (Kristensen et al., 2018).

**FIGURE 1:**
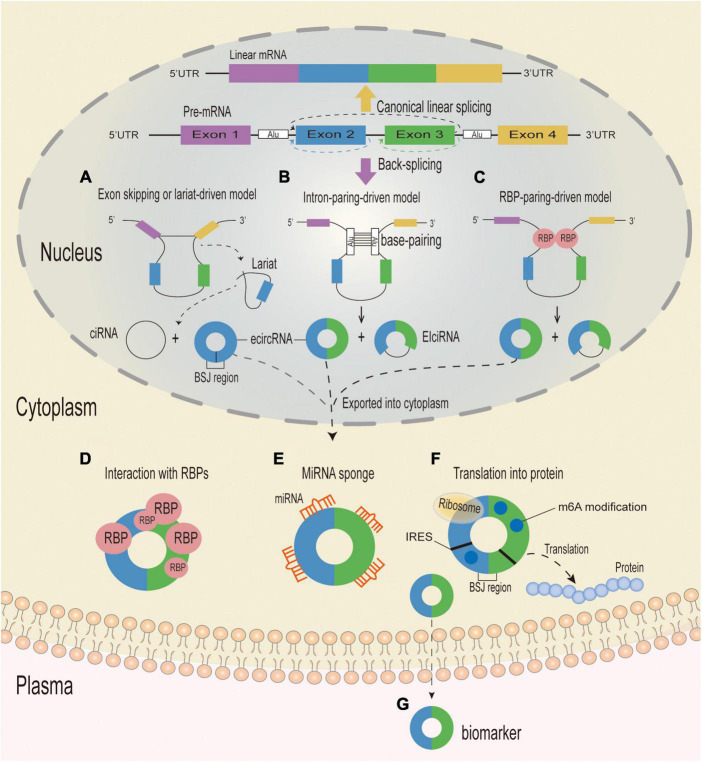
Biogenesis mechanisms and putative functions of circRNA. Eukaryotic pre-mRNAs can be spliced to generate linear or circular RNA. When splice sites are joined in a linear order by the pre-mRNA splicing machinery, a canonical linear mRNA is generated (Yellow arrow). Alternatively, back-splicing can join a 5′ splice site to an upstream 3′ splice site, resulting in the production of a circular RNA whose ends are covalently linked by a 3′–5′ phosphodiester bond (Purple arrow). **(A)** Exon-skipping or lariat-driven circularization. In the exon-skipping or lariat-driven model, an exon skipping event results in covalently splicing from the 3′ splice donor site to the 5′ splice acceptor site, which forms an RNA lariat structure containing the exon and intron and a linear product (not shown). At the same time, the introns are removed by the spliceosome to form an ecircRNA, and the intronic lariat precursors which escape from the debranching step of canonical linear splicing can form a ciRNA. **(B)** Direct back-splicing or intron-pairing-driven circularization. In the intron-pairing-driven model, direct base-paring of the complementary sequence motifs (such as Alu elements) forms a circulation structure and a linear product (not shown), and then the introns are removed or retained to form an ecircRNA or an EIciRNA. **(C)** RBP-driven circularization. In the RBP-pairing-driven model, the interaction between two RBPs can bridge two flanking introns together and form a circulation structure and a linear product (not shown), and then the introns are removed or retained to form an ecircRNA or an EIciRNA. CircRNA can be divided into three different types: ecircRNA, ciRNA and EIcRNA. CiRNAs and EIcRNAs are likely sequestered in the nucleus, while ecircRNAs are mostly exported into the cytoplasm and can play a variety of functions. **(D)** CircRNAs with RBP-binding motifs may function as sponges or decoys for proteins and thereby regulate their activity. **(E)** CircRNAs containing miRNA response elements might regulate expression of miRNA-target mRNAs through sequestering or sponging with the miRNAs. **(F)** CircRNAs containing IRES and/or with m6A modifications can serve as templates for translation and give rise to circRNA-specific peptides when the reading frame extends across the BSJ. **(G)** CircRNAs can be excreted into the extracellular space, such as plasma, and function as biomarkers. UTR, untranslated region; RBP, RNA-binding-protein; BSJ, back-splice junction; circRNA, circular RNA; ciRNA, circular intronic RNA; EIciRNAs, exon–intron circRNAs; ecircRNA, circular exonic RNA; miRNA, microRNA; IRES, Internal Ribosome Entry site; m6A, N6-methyladenosines.

The existing proposed biogenetic models of circRNA synthesis mainly consist of the following: exon-skipping or lariat-driven circularization, intron pairing-driven circularization, intronic circRNA (ciRNA) biogenesis, and RNA-binding protein(RBP)-associated pairing-driven circularization (Zhang et al., 2013; Li et al., 2015). Circularization begins with an exon-skipping event in the lariat-driven circularization model, which requires a covalent interaction between the 5′ and 3′ splice sites to generate a lariat precursor that contains exons for efficient circle production (Figure 1A; Jeck and Sharpless, 2014; Barrett et al., 2015; Lee and Rio, 2015). In the intron pairing-driven circularization model, circularization happens through direct base pairing between reverse complementary sequences—with introns containing Alu repeats being more probable to pair—resulting in exon circularization and the production of several types of circRNAs (Figure 1B; Zhang et al., 2014). CircRNAs can also be made from lariat precursors that do not get broken up by standard linear splicing (Zhang et al., 2013). Quaking and muscleblind are two RBPs that play a role in pairing-driven circularization. The RBP-associated process is activated by the close proximity of circRNA-forming exon and intron splice sites, which is made possible in introns by the complementary base pairing of inverted repeats (Figure 1C; Ashwal-Fluss et al., 2014; Conn et al., 2015).

Circular RNAs can be classified into three groups based on their components, circularization mechanism, origin, and genomic organization: exonic circRNAs, which are produced from a single or many exons, account for over 80% of circRNAs, and are predominantly found in the cytoplasm (Gao et al., 2016); exon–intron circRNAs (EIciRNAs), which are created from exons and introns and are mostly localized in the nucleus (Li et al., 2015); and ciRNAs, which are derived from introns (Kristensen et al., 2019). Recent research has revealed three novel forms of circRNAs: transfer RNA (tRNA) intronic circRNAs, fusion circRNAs, and mitochondria-encoded circRNAs. While the majority of circRNAs are synthesized from precursor mRNA, tRNA intronic circRNA is synthesized from precursor tRNA. The tRNA splicing endonuclease complex can cleave a precursor tRNA with introns, and the intron ends can then be cyclized by the RtcB ligase, resulting in a mature tRNA and tRNA intronic circRNA (Schmidt et al., 2019). Fusion circRNAs contain two intronic circRNA fragments that are flanked by GT–AC splicing signals and can be detected using CircRNA Identifier (CIRI, a novel circRNA identification algorithm) (Gao et al., 2015). It was recently demonstrated that mitochondria-encoded circRNA is synthesized in a splicing-independent manner and acts as a molecular chaperone, facilitating the entry of nuclear-encoded proteins into the mitochondria (Liu et al., 2020b).

## Detection and analysis methods of circRNAs

It is challenging to detect circRNA because their sequences are almost identical to the sequences of their linear cognate RNAs. Current methods to detect and quantify circRNAs include high throughout RNA-seq, circRNA microarray, and reverse transcription-PCR (RT-PCR)/quantitative RT-PCR (qRT-PCR) using divergent primers and northern blot over the back-splice junction (BSJ) sites.

Reverse transcription-PCR/quantitative RT-PCR detection ensures that the detected circRNAs are based on primer specificity and site design, which is generally used for data validation after high-throughput sequencing and quantitative detection in subsequent functional studies. Convergent primers serve as a reference for the amplification of either genomic or linear RNA or circRNAs, whereas divergent primers are only circRNAs. But RT-PCR/qRT-PCR does not guarantee the existence of a circular RNA. Because divergent PCR is commonly used to validate circRNAs by amplifying speculative BSJ sites; however, it can also amplify linear RNA with the same BSJ sequence locus.

Northern blotting is another easy and effective method for detecting circRNA (Capel et al., 1993). Probes are designed to selectively target the diagnostic junctional sequence as well as the circularized exonic sequence. So far, northern blotting is the most accurate approach for detecting the abundances and sizes of circRNA, though its sensitivity is poor.

Circular RNA microarray technique captures and quantifies circRNA with high sensitivity and specificity by combining known circular junction sequence-specific probes with linear RNA depletion by exonucleases (Jeck et al., 2013). However, it can only detect known circRNAs, and the results should be validated by other methods.

Circular RNA can be identified on a large scale by high throughout RNA-Seq technology. Since the proportion of circRNA in total RNA is relatively low, circRNA is generally enriched in order to increase the types and detection effect of circRNA. To determine whether RNA is actually circular and to enrich for circRNA in sequencing libraries, RNase R, an exoribonuclease that can processively degrade RNA from its 3′ to 5′ end, is a valuable tool that is frequently used in conjunction with other methods such as RT-PCR and northern blotting (Venø et al., 2015). But RNase R is not absolutely unable to digest circRNA. Too long a digestion time and too high a dosage of RNase R may lead to a significant reduction of circRNA content after enzyme digestion. Besides, Sanger sequencing verification of the BSJ’s existence is yet another essential part in the validation of circRNAs.

The approaches for investigating circRNA are maturing as we gain better knowledge of it. In addition to Ribo-RNA-seq and RNase R-treated RNA-seq, sequencing data can be examined for whole-genome detection and quantification (Zhang et al., 2016; Dong et al., 2017). Northern blotting or RT-qPCR can then be used to confirm the results. Typically, nuclear-cytoplasm fractionation or fluorescence *in situ* hybridization were used to determine subcellular localization (Memczak et al., 2013; Li et al., 2015). The association between circRNA and target genes was confirmed using RNA immunoprecipitation assays (Schneider et al., 2016; Chen et al., 2019b). For functional validation, a silencing model was built with small interfering RNA (siRNA) or short hairpin RNA (shRNA) (Guarnerio et al., 2016; Chen et al., 2019a), and an overexpression model was built with plasmid or adenovirus (Wang et al., 2016; Litke and Jaffrey, 2019).

In addition to the biochemical methods mentioned above, there are many circRNA-associated public databases that can provide predictions on circRNAs’ possible biological functions or regulatory network in human diseases, including StarBasev2.0 (Li et al., 2014) and circInteractome (Dudekula et al., 2016) (which predict the circRNA-miRNA-mRNA network and interaction between circRNA and RBPs), CircRNADb (Chen et al., 2016), and CSCD (Xia et al., 2018) (which predict the protein-encoding ability of circRNAs), and circ2Trait (Ghosal et al., 2013) (which provides information on 1951 human circRNAs and their association with 105 human diseases). But the lack of standardized nomenclature makes it difficult to search the same circRNA in different databases, and the curated database is still limited.

## Functions of circRNAs

Circular RNAs were originally thought to be by-products of abnormal RNA splicing or genomic noise (Sanger et al., 1976; Cocquerelle et al., 1993). However, with the rapid advancement of RNA sequencing (RNA-seq) technologies, an increasing number of studies have established that circRNAs are not the meaningless by-products of mis-splicing, but rather are regulatory biomolecules involved in a variety of physiological and pathological processes. CircRNAs perform many different roles, including serving as miRNA sponges (Hansen et al., 2013), controlling transcription or splicing (Chen, 2016), interacting with RBPs (Conn et al., 2015), translating proteins (Legnini et al., 2017), and acting as protein decoys or scaffolds (Figures 1D–G; Du et al., 2016).

## MiRNA sponging

MicroRNAs are a class of ubiquitous, conserved small ncRNAs with a length of 19–22 nucleotides. They can act as transcriptional regulators via base-pairing directly to target sites in the 3′ untranslated regions (UTRs) of mRNA, thereby negatively regulating mRNA expression and eventually resulting in decreased mRNA stability and translation suppression. According to the competing endogenous RNA (ceRNA) theory, additional RNAs containing miRNA target sites can compete with mRNAs for miRNA binding (Tay et al., 2014). Multiple studies have reported that many circRNAs contain a variety of miRNA response elements that precisely bind to the matching miRNAs to inhibit their activity while increasing the production of miRNA target molecules (Liu et al., 2017). The most prevalent and conspicuous function of circRNAs is as miRNA sponges. The cerebellar degeneration-related protein 1 gene (CDR1) is the most well-known circRNA to act as a ceRNA; it is extensively expressed in both human and mouse brains and contains over 60 conserved miR-7 binding sites. By acting as a strong miRNA sponge for miR-7, CDR1 antisense (CDR1as) reduces miR-7 levels and indirectly increases the expression of miR-7 target genes, thus impairing zebrafish midbrain development and regulating the processes of many diseases (Guo et al., 2020).

## Transcriptional regulation

While the majority of circRNAs are mainly located in the cytoplasm, EIciRNAs and ciRNAs are primarily located in the nucleus and induce the transcription of their parental genes. Both EIciRNAs and ciRNAs act as *cis*-regulators to influence parental gene expression. The EIciRNAs–U1 small nuclear ribonucleoprotein (U1 snRNP) complex is formed when EIciRNAs connect to the U1 snRNP, which subsequently interacts with polymerase II (Pol II) in the host gene transcription promoter region, thereby increasing the expression of their parental genes (Li et al., 2015). Similarly, by influencing the elongation of Pol II, ciRNAs can cluster at their synthesis sites and regulate parental genes by exerting *cis*-regulatory effects (Zhang et al., 2013). Cir-EIF3J and circ-PAIP2 are two examples of EIcircRNAs; they can both interact with U1 snRNP in the nuclei of 293T and HeLa cells to enhance the transcription of their parental genes (Li et al., 2015). Zhang et al. (2013) observed that ci-ANKRD52 binds selectively to the elongation RNA Pol II complex and directly increases *ANKRD52* transcription. However, inhibiting ci-ANKRD52 decreased the *ANKRD52* transcription rate. Together, these findings suggest that unique RNA–RNA interactions represent a distinct transcriptional control mechanism.

## Translation into proteins

Ribosomes carry out the translation process by initiating, elongating, terminating, and recycling ribosomes (Schuller and Green, 2018), and the translation of eukaryotic mRNAs is dependent on the 5′-cap structure (known as cap-dependent translation). Because circRNAs lack a 5′ cap and a poly (A) tail, they were formerly believed to be unique endogenous ncRNAs and were deemed untranslated. However, recent research suggest that some circRNAs in the cytoplasm may be capable of encoding proteins in a cap-independent manner (Pamudurti et al., 2017; Lei et al., 2020). For example, emerging evidence suggests that internal ribosome entry sites within the sequence or N^6^-methyladenosine in the 5′-UTR of circRNAs are likely to have high translational potential (Meyer et al., 2015). Thus, cap-independent initiation of translation via internal ribosome entry sites and N^6^-methyladenosine may be viable mechanisms for circRNA translation, and these two methods may also be combined (Chen and Sarnow, 1995; Yang et al., 2017). Several endogenous circRNAs have been shown to function as translation templates for protein encoding when internal ribosome entry site elements drive them or N^6^-methyladenosine RNA modifies them. As an example, circ-ZNF609 can act as a template for translation in a splicing-dependent and cap-independent manner, and can be translated into a protein (Legnini et al., 2017). Besides, the peptide PINT87aa coded by circPINT exon 2 interacts directly with the Pol II-associated factor 1 (PAF1) complex, thus inhibiting oncogene expression and restricting transcriptional elongation (Zhang et al., 2018).

## Interactions with RBPs

RNA-binding proteins are a group of proteins with RNA recognition patterns that help with RNA maturation, transportation, localization, and translation (Qin et al., 2020). They can also regulate RNA metabolic activities via RNA binding (Gebauer et al., 2021), and a lack or malfunction of RBPs can lead to the development of a range of disorders. CircRNAs specifically bind to RBPs either directly or indirectly via RNA to regulate gene expression (Du et al., 2017). For example, HuR (or ELAVL1) is a well-characterized RBP that suppresses *p27* translation in the 5′-UTR of *p27* via an interferon-responsive sequence region (Kullmann et al., 2002); a recent study indicated that circBACH1 can interact with HuR to increase its translocation and cytoplasmic accumulation, thereby suppressing *p27* expression (Liu et al., 2020a).

## Protein scaffolding

The function of circRNAs as protein scaffolds means that circRNAs harboring binding sites for enzymes and their substrates are likely to serve as dynamic scaffolds mediating protein-protein interactions to assemble large RNA-protein complexes. This is perhaps best exemplified by circFoxo3, which is highly expressed in the cytoplasm of mouse non-cancerous cells and is connected to cell cycle progression. By interacting with the cell cycle proteins cyclin-dependent kinase 2 (CDK2) and cyclin-dependent kinase inhibitor 1 (p21), it forms the circFoxo3-p21-CDK2 ternary complex. The formation of the circFoxo3-p21-CDK2 ternary complex arrests the function of CDK2, which is normally required for cell cycle progression. Several complimentary approaches, including circRNA pulldown with targeted probes, siRNA, and RNase A protection assays, were used to validate this ternary complex (Du et al., 2016). Similarly, circACC1 directly binds to β and γ subunits of AMP- activated protein kinase (AMPK) to form a ternary complex, stabilizing and promoting the enzymatic activity of the AMPK holoenzyme, thus playing a role in metabolic adaptation to serum deprivation (Li et al., 2019).

## CircRNAs and PD

**FIGURE 2:**
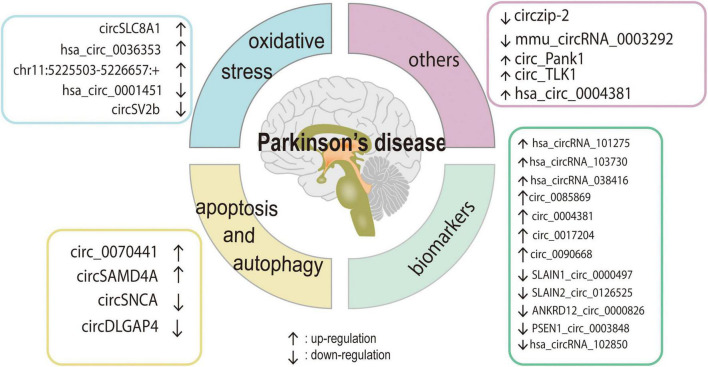
The role and regulatory pathway of PD-related circRNAs. Various circRNAs could participate in the development of PD by affecting cell apoptosis, autophagy, oxidative stress, and some of them may be the potential biomarkers for PD.

Parkinson’s disease is the second most prevalent neurodegenerative disease in middle-aged and older people and is defined by a marked reduction in striatal DA content that is caused by the degeneration and death of DA neurons in the SN of the midbrain (Falkenburger et al., 2016). Recent research has demonstrated that the pathophysiology of PD is complicated by mitochondrial malfunction, excessive oxidative stress, aberrant cell apoptosis, and ubiquitin-proteasome system dysfunction. Furthermore, emerging evidence suggests that circRNAs play a role in the development of PD by influencing α-syn expression, oxidative stress, aberrant transcription regulation, cell apoptosis, and autophagy (Kumar et al., 2018; Feng et al., 2020; Hanan et al., 2020; Jia et al., 2020; Wang et al., 2021b). Here, we will discuss the roles of circRNAs in the regulation of various cellular activities. The circRNAs that have been identified as dysregulated in PD are summarized in Table 1 and Figure 2.

**TABLE 1:** Dysregulated circRNAs in Parkinson’s disease.

Mechanisms	CircRNAs	Sample types	Expression	Sponge targets
Apoptosis and autophagy	circDLGAP4	Cell lines, mouse	Down	miR-134-5p/CREB
	circSAMD4A	Cell lines, mouse	Up	miR-29c-3p/AMPK/mTOR pathway
	circSNCA	Cell lines	Down	miR-7
	circ_0070441	Cell lines	Up	miR-626/IRS2
Oxidative stress	circSLC8A1	Brain tissue	Up	miR-128
	hsa_circ_0036353	Blood	Up	–
	chr11:5225503-5226657: +	Blood	Up	–
	hsa_circ_0001451	Blood	Down	–
	CircSV2b	Mouse	Down	miR-5107-5p/Foxk1/Akt1
α-syn metabolism	circzip-2	*C. elegans*	Down	miR-60-3p
Neuroinflammation Others	circ-Pank1	Cell lines, mouse	Up	miR-7a-5p/α-syn
	hsa_circ_0004381	Cell lines	Up	miR-185-5p/RAC1
	circTLK1	Cell lines, mouse	Up	miR-26a-5p/DAPK1
	mmu_circRNA_0003292	Mouse	Down	miRNA-132/Nr4a2

α-syn, alpha-synuclein; CREB, cAMP-response element binding protein; IRS2, Insulin Receptor Substrate 2; DAK1, death-associated protein kinase 1.

## CircRNAs involved in apoptosis and autophagy

Accumulating evidence suggests that autophagy and apoptosis play an important role in the pathogenesis of PD. Autophagy dysregulation impairs many cellular processes, including α-syn degradation (Lu et al., 2020), and while apoptosis is required for neural network formation, excessive apoptosis accelerates the progression of PD (Li et al., 2020a). Although apoptosis is primarily responsible for the death of DA neurons in PD, aberrant apoptosis is also a sign of DA depletion in the substantia nigra, which has a significant impact on the onset of PD (Liu et al., 2019). Furthermore, circDLG-associated protein 4 (circDLGAP4) expression is reportedly downregulated in a 1-methyl-4-phenyl-1,2,3,6-tetrahydropyridine hydrochloride (MPTP)-induced PD mouse model as well as in 1-methyl-4-phenylpyridinium (MPP^+^)-treated PD cell lines (Feng et al., 2020). In an *in vitro* study, circDLGAP4 knockdown resulted in mitochondrial damage, reduced autophagy, and increased apoptosis in SH-SY5Y and MN9D cells, whereas circDLGAP4 overexpression reduced the effects of MPP^+^. Further research revealed that circDLGAP4 acts as a miR-134-5p sponge. Because miR-134-5p directly targets the cAMP-response element binding protein (CREB), circDLGAP4/miR-134-5p may also modulate CREB signaling activation and thus promote the expression of CREB target genes such as B-cell lymphoma 2, peroxisome proliferator-activated receptor gamma coactivator 1-α, and brain-derived neurotrophic factor. These findings imply that the circDLGAP4/miR-134-5p/CREB axis may contribute to the development of PD (Feng et al., 2020).

Wang et al. discovered that the circular RNA sterile alpha motif domain containing 4A (circSAMD4A) was increased in both animal and cell models of PD (Wang et al., 2021b). Cellular and animal experiments confirmed that in MPTP- or MPP^+^-induced PD models, circSAMD4A knockdown not only suppressed apoptosis and autophagy, thus lowering MPTP or MPP^+^ cellular toxicity, but also suppressed phosphorylated-AMPK expression and elevated mTOR expression. A miR-29c-3p inhibitor reversed all of the aforementioned effects. Moreover, additional research revealed that circSAMD4A directly targeted and adversely regulated miR-29c-3p. Together, these findings indicate that circSAMD4A alters the AMPK/mTOR cascade via miR-29c-3p, thus resulting in apoptosis and autophagy of DA neurons in PD; circSAMD4A may therefore be an effective therapeutic target for PD (Wang et al., 2021b).

After treatment with pramipexole, Sang et al. found that circSNCA was downregulated in an MPP^+^ induced SH-SY5Y cell model of PD (Sang et al., 2018). Both SNCA and genes that promote apoptosis showed the same trend as circSNCA, whereas genes known to inhibit apoptosis and autophagy showed the opposite trend. A bioinformatic study revealed that both circSNCA and SNCA 3′-UTR target the same part of the miR-7 seed region; double luciferase reporter experiments and immunofluorescent localization experiments confirmed this finding. As a result, the authors hypothesized that *SNCA* expression is upregulated by functioning as a miR-7 sponge and that circSNCA downregulation via pramipexole therapy might decrease cell apoptosis and enhance cell autophagy in PD (Sang et al., 2018). Hsa_circ_0070441, a circRNA generated from the mRNA coding sequence region of *SNCA*, is reportedly upregulated in MPP^+^-induced SH-SY5Y cells (Cao et al., 2022). By sponging miR-626, knocking down hsa_circ_0070441 reduces MPP^+^-induced inflammation and neuronal apoptosis in SH-SY5Y cells. Additionally, miR-626 has been reported to target insulin receptor substrate 2. These findings imply that hsa_circ_0070441 may exacerbate MPP^+^-induced neurotoxic effects in SH-SY5Y cells via the miR-626/insulin receptor substrate 2 axis and may thus be a potential diagnostic target for PD (Cao et al., 2022).

## CircRNAs in oxidative stress

Convincing evidence indicates that oxidative stress, which results from an imbalance between oxidant production and antioxidative defenses (Wang et al., 2020; Forman and Zhang, 2021), has a notable effect on the pathological progression of PD. Because of their high oxygen consumption and low concentrations of antioxidant enzymes, neurons are susceptible to oxidative stress damage. To date, increasing research indicates that oxidative stress plays an important role in both the etiology and progression of PD (Wang et al., 2020; Zhang et al., 2022).

It is well established that oxidative reagents such as paraquat may increase an individual’s probability of developing PD (Berry et al., 2010). Hanan et al. (2020) used RNA-seq to profile circRNAs in three brain regions—the SN, middle temporal gyrus, and amygdala—from dozens of PD patients and healthy people, and reported enhanced circSLC8A1 expression in the SN of PD patients. To determine whether oxidative stress has a direct effect on circSLC8A1 levels, they next exposed cultured human SH-SY5Y neural cells to increasing doses of paraquat. Surprisingly, paraquat exposure dose-dependently increased circSLC8A levels, but these levels were reduced in cells treated with the neuroprotective drug simvastatin and the LRRK2 inhibitor PF-06447475 (Hanan et al., 2020).

More recently, Kong et al. (2021) identified a large number of differently expressed circRNAs between PD patients and healthy controls using RNA-seq for peripheral blood RNAs. The top two upregulated circRNAs in the PD group were hsa_circ_0036353, encoded by *SIN3A*, and chr11:5225503-5226657: +, a circRNA encoded by *HBB* that had not previously been identified. In addition, one of the top eight downregulated circRNAs was hsa_circ_0001451, encoded by *FBXW7*. Notably, S*IN3A*, *HBB*, and *FBXW7* are all involved in the oxidative stress response (Kong et al., 2021). Unfortunately, the results of this study were derived from bioinformatic analysis alone; additional experimental studies are required to validate these findings.

Cheng et al. (2022) performed ribosomal RNA-depleted RNA-seq on SN pars compacta and striatum tissue from a PD mouse model as well as paired control tissue and found that circSV2b was downregulated. They then used a bioinformatic database to predict the target miRNAs of circSV2b as well as its downstream mRNAs and discovered that miR-5107-5p and *Foxk1* were the targeted factors of circSV2b and miR-5107-5p, respectively (Cheng et al., 2022). Because oxidative stress plays a crucial role in the cascade leading to DA cell degeneration in both familial and sporadic PD, they further screened for genes associated with oxidative stress downstream of *Foxk1* and identified that *Akt1* (an intermediate regulatory molecule of Foxk1) was involved in PD. Their mechanistic analysis demonstrated that circSV2b overexpression leads to reduced oxidative stress damage through the miR-5107-5p/*Foxk1*/*Akt1* axis in PD models (Cheng et al., 2022).

Together, the findings from these studies clearly suggest that circRNAs may have a role in the etiology of PD via an oxidative response.

## CircRNAs as potential biomarkers in PD

Despite advances in neuroimaging and genetics, PD is predominantly diagnosed clinically by the presence of at least two neuromotor symptoms, such as resting tremor, bradykinesia, rigidity, and/or postural instability (Armstrong and Okun, 2020). But phenotypic similarities to other atypical parkinsonian disorders, such as multisystem atrophy or corticobasal degeneration, complicate the diagnosis of PD; the symptoms of these disorders overlap with those of PD, particularly in its early stages. As a consequence, accurately diagnosing PD is extremely challenging, and there is a high risk of misdiagnosis during the early stages of the disease. In this context, the identification of a distinct biological marker that can be obtained using non-invasive procedures is critical for the early detection of PD.

Circular RNAs are relatively tolerant of RNA exonuclease and have high stability in blood and other bodily fluids because of their covalently closed ends (Memczak et al., 2013). Additionally, the brain and exosomes have more circRNAs than other tissues, and their abundance and conservation can be accurately assessed using conventional and rapid laboratory procedures, such as real-time reverse transcription polymerase chain reaction. These features make circRNAs attractive candidates for biomarkers of neurological disorders. Indeed, circRNAs have been identified in blood, urine, cerebrospinal fluid, exosomes, and extracellular vesicles (Cheng et al., 2020; Tran et al., 2020), and have been recommended as biomarkers for cancer and a variety of other diseases (Ostolaza et al., 2020; Zaiou, 2020).

Eighty-seven circRNAs with comparatively high gene expression in the human brain were identified by Ravanidis et al. (2021), who then examined their expression in peripheral blood mononuclear cells from 60 idiopathic PD patients and 60 healthy individuals. Six circRNAs were significantly downregulated in idiopathic PD patients compared with healthy controls. The authors then performed a receiver operating characteristic curve analysis to determine the utility of peripheral blood mononuclear cell circRNA levels for differentiating subjects with idiopathic PD from healthy control subjects. The diagnostic sensitivity and specificity of a four-circRNA panel (SLAIN1_circ_0000497, SLAIN2_circ_0126525, ANKRD12_circ_0000826, and PSEN1_circ_0003848) were 75.3 and 78%, respectively, and the area under the curve was 0.84. These findings indicate that the four-circRNA panel had acceptable sensitivity and specificity for idiopathic PD in this discovery group, which suggests that the panel may be used to diagnose and treat PD.

Similarly, a recent study discovered elevated levels of circ_0017204, circ_0085869, circ_0004381, and circ_0090668 in plasma samples taken from people with PD. Correlation analysis revealed that the circ_0017204 and circ_0004381 panels may be able to accurately differentiate individuals with early-stage PD from healthy controls, whereas the circ_0085869, circ_0004381, circ_0017204, and circ_0090668 panels may be able to differentiate the late stages of PD from the early stages and thereby serve as a dynamic monitoring factor for PD progression (Zhong et al., 2021).

In our previous work, we used microarray analysis to investigate the global expression levels of circRNAs in PD patients and controls and then verified the candidate circRNAs in another PD cohort. Compared with controls, hsa_circRNA_101275, hsa_circRNA_103730, and hsa_circRNA_038416 had significantly higher expression in PD patients, and hsa_circRNA_102850 had lower expression in PD patients. A circRNA panel containing the four differentially expressed circRNAs had a strong diagnostic capacity (area under the curve = 0.938) for distinguishing PD patients from controls (Xiao et al., 2022).

## CircRNAs in α-syn metabolism

The primary pathological sign of PD is α-syn aggregation in the cytoplasm of DA neurons in the SN, and accumulating data indicate that α-syn plays a critical role in both the onset and progression of PD. Correlation analyses have also revealed a link between α-syn, neurotoxicity, and the anti-apoptotic pathway (Junn et al., 2009). In some families with autosomal dominant early-onset PD, point mutations and gene duplication or triplication events have been identified in the α*-syn* locus. According to one study, α-syn is a target of miR-7, and neuronal miR-7 levels are linked to α-syn accumulation and aggregation both *in vitro* and *in vivo* (McMillan et al., 2017). Notably, circRNAs can also be produced by the gene that encodes α*-syn* mRNA. CircSNCA, for example, is derived from the proximal 3′UTR of α*-syn* mRNA (Sang et al., 2018), and it can effectively sponge miR-7 in cell culture. Hsa_circ_0070441 is also derived from the coding sequence region of α*-syn* mRNA (Cao et al., 2022). These data suggest that genetic mutations may also be associated with circRNAs.

Kumar et al. (2018) reported that circzip-2 expression is markedly downregulated in the transgenic *Caenorhabditis elegans* NL5901 model of PD, which expresses human α-syn protein in the body wall muscle. Using the bioinformatics tool miRbase, they discovered 11 miRNAs as putative circzip-2 interacting miRNAs; miR-60-3p was the sole miRNA with confirmed mRNA targets among the detected 11 molecules (Kumar et al., 2018). These findings point to a possible relationship between circzip-2, miR-60-3p, and α*-syn*. Regrettably, however, these authors did not conduct any studies to elucidate the underlying mechanisms of such interactions.

Circ-pantothenate kinase 1 (Pank1) is a circRNA derived from the Pank1 gene and was highly expressed in both the SN of PD model mice treated with rotenone and in a MN9D cell model of DA neurons (Liu et al., 2022). Further investigation revealed that the mRNA and protein expression levels of α*-syn* in MN9D cells treated with rotenone were also significantly increased. To address the hypothesis that circ-Pank1 upregulates α*-syn* expression, the researchers used small interfering RNAs targeting the back-splice junction sites of circ-Pank1 and transfected them into MN9D cells treated with rotenone. They found that the knockdown of circ-Pank1 decreased the expression levels of α*-syn* protein as well as α*-syn* mRNA. As described in the miRNA sponge section, the most prevalent and conspicuous functions of circRNAs are as miRNA sponges to regulate downstream genes. The authors, therefore, used online bioinformatic databases to identify potential miRNAs that target α*-syn* mRNA sponged by circ-Pank1, and miR-7a-5p was predicted. Luciferase reporter assays confirmed that circ-Pank1 functions as an efficient miR-7a-5p sponge and that miR-7a-5p targets α*-syn* expression. Mechanism research revealed that circ-Pank1 regulates DA neuronal injury through the targeted regulation of the miR-7a-5p/α*-syn* axis. This study described a novel circRNA-α*-syn* regulatory mechanism and identified a potential therapeutic target for PD (Liu et al., 2022).

## CircRNAs in neuroinflammation

Chronic inflammation is one of the hallmarks of PD pathophysiology, and studies of animal models of PD have also identified neuroinflammation as a critical contributor to PD progression. Activated microglia generate a significant number of proinflammatory cytokines during neuroinflammatory reactions, which ultimately lead to DA neuronal apoptosis (Troncoso-Escudero et al., 2018). In the SN of PD patients, the expression of astrocytes, activated microglia, and proinflammatory mediators is increased (Xin and Liu, 2021). Moreover, studies have reported that circRNA is associated with neuroinflammation. For example, the knockdown of circ-0000220 or overexpression of its target miR-326-3p in BV-2 microglial cells lowers the production of inflammatory cytokines (Li et al., 2020b). Circ-001372 reduces inflammation in a propofol-induced neurotoxicity and neuroinflammation rat model and decreases neural apoptosis through PIK3CA/Akt/NF-kappaB via miRNA-148b-3p (Wang et al., 2021a).

## Other dysregulated circRNAs in PD

Using RNA-seq, a recent study identified the transcriptome patterns of circRNAs in the hippocampus, cerebellum, cerebral cortex, and striatum of an MPTP-induced animal model of PD. In this investigation, they discovered 66, 24, 121, and 71 different circRNAs in the hippocampus, cerebral cortex, cerebellum, and striatum, respectively, compared with the control group. Further analysis of the expression levels of the identified circRNAs indicated that the mmu_circRNA_0003292/miRNA-132/Nr4a2 pathway may be implicated in the molecular mechanism of PD (Jia et al., 2020). Moreover, Chen et al. suggested that circTLK1 may act as an endogenous sponge of miR-26a-5p to inhibit its function, thus leading to the increased expression of death-associated protein kinase 1 and the consequent exacerbation of neuronal injury (Chen et al., 2022).

## CircRNAs in aging

The single most important independent risk factor for the onset of PD is aging. The majority of human variance in aging is believed to be caused by lifestyle and environmental variables, with genes likely accounting for about 25% of the variation (Jin et al., 2020). Investigations of the association between circRNAs and human aging have focused on the potential of circRNAs as biomarkers of aging and age-related disorders; numerous circRNAs have been identified with levels that are altered in an age-dependent manner across particular human organs or tissues. For example, aged humans have higher levels of circ_1305 and circ_722 but lower levels of circ_1445 than young people (Dluzen et al., 2018), and circDEF6, circFOXO3, circEP300, and circFNDC3B are associated with aging phenotypes in humans (Haque et al., 2020). Besides, Alzheimer’s disease and other age-related neurodegenerative diseases have been linked to circRNAs. In 2013, a unique circRNA was found in astrocytes in the posterior cingulate cortex of Alzheimer’s disease patients but not in those of disease-free control samples (Haque et al., 2020). Physiological changes in DA neurons in the SN are also correlated with age-dependent variations in circRNA expression, and 98 circRNAs have been identified to have differential expression in the SN of elderly rats using RNA-seq analysis (Sekar et al., 2018). Overall, however, circRNA-related aging research remains in its infancy, and further research is required to determine whether these circRNAs are differentially expressed in aging brain regions or are linked to age-related neurodegenerative diseases, including Alzheimer’s disease and PD.

## Conclusions and perspectives

Parkinson’s disease is a multifactorial and multistage neurodegenerative disease, and the precise pathogenic pathways that cause this disease are not yet fully understood. Despite the application of comprehensive treatment programs that include surgery, medications, rehabilitation, and exercise therapy, the treatment of PD remains unsatisfactory. The identification of more efficient therapeutic techniques is therefore critical for the clinical management of PD. CircRNAs, which were traditionally thought to be by-products of RNA splicing mistakes, have gained increasing attention in recent years because of their strong associations with a variety of physiological and pathological processes (Geng et al., 2018). Numerous studies have signaled the utility of circRNAs in the clinical treatment of a range of neurodegenerative diseases, including PD. As discussed throughout the present review, circRNAs are abnormally expressed in PD and are involved in a variety of biological processes associated with the disease, including apoptosis, autophagy, and oxidative stress. They are thus promising biomarkers for PD diagnosis and treatment.

Circular RNA research in PD is in its infancy compared with that of miRNA and long ncRNA; only a small subset of significant circRNAs have been identified and characterized in PD. Moreover, in the field of circRNAs in PD, several points must be addressed in the future. First, circRNAs have primarily been found to exert their effects on PD through their function as miRNA sponges. It is thus critical to explore the additional activities of circRNAs in PD, including their translational capacity, gene transcription control, and interaction with RBPs. Second, because of their unique circular structure and resistance to the exonuclease RNase R, circRNAs are kept in reasonably stable conditions in cells and can survive for an extended period of time in the extracellular environment. This means that the identification of circulating circRNAs that are aberrantly expressed in body fluids may be effective for disease detection. To date, circRNAs have been discovered to function as biomarkers of malignancies in clinical tissues such as serum, plasma, and exosomes. However, in PD, there is as yet no research on circulating circRNAs from saliva, urine, cerebrospinal fluid, exosomes, or other sources; these should be investigated in future research. Third, current studies have mainly focused on a specific circRNA and its downstream mechanisms, whereas the upstream mechanisms (such as how circRNAs are exported from the nucleus to the cytoplasm or how they are formed) remain largely unknown. Even in the most recent research, these difficulties are scarcely acknowledged. Finally, because the ultimate goal of circRNA research in PD is to be able to apply specific disease-associated circRNA findings to the therapy of PD, more controlled clinical investigations and experiments in PD patients are required.

The well-established functional roles of circRNAs in tumorigenesis identify them as obvious targets for anticancer therapy. By using CRISPR–Cas9 (Zheng et al., 2016) systems, CRISPR–Cas13 (Zhang et al., 2021) systems, or target the circRNA for destruction (Ottesen et al., 2019), oncogenic circRNAs can be selectively inhibited or degraded. Moreover, the overexpression of natural tumor-suppressor circRNAs or the expression of synthesized circRNAs that contain tumor-suppressive elements may be novel therapies for cancer. For example, the most popular strategy in preclinical investigations involves delivering circRNAs with repetitive miRNA-binding sites, which can serve as ceRNAs for oncogenic miRNAs (He et al., 2021). But there are currently no circRNA-targeted medicines in clinical trials, and circRNAs for gene therapy almost exclusively focus on preclinical animal models.

As a genetically related disease, gene mutation is a key factor in the etiology of PD, and recent studies have confirmed that gene therapy can be effective in PD. The most effective treatment for motor symptoms in PD is levodopa, but to convert levodopa to dopamine, L-amino acid decarboxylase (AADC) is required (Christine et al., 2019). NBIb-1817 (VY-AADC01) is an experimental adeno-associated virus serotype 2 gene therapy that encodes the human AADC enzyme, and it can increase dopamine production by delivering the AADC gene directly to the putamen. In the 36-month safety and clinical outcomes of the PD-1101 trial of VY-AADC01, VY-AADC01 was able to be infused into the bilateral putamen of PD patients using intraoperative MRI-guided technology. This reduced the requirements for PD medications by 21–30% and stabilized or improved standard measures of motor function, global impressions of improvement, and quality of life in PD patients compared with the baseline (Christine et al., 2022). It is worth noting that circRNAs have been found to influence the progression of PD by regulating multiple cellular processes, which makes them potential therapeutic targets. Moreover, gene therapy based on circRNA is likely to provide a broader prospect for the precision treatment of PD.

In summary, the present review detailed the biogenesis, properties, and roles of circRNAs in PD. Despite having a limited knowledge of functional circRNAs in PD, we anticipate that with the advent of novel bioinformatic analytical tools, a better understanding of the characteristics of circRNAs will be gained, and circRNAs may be used as diagnostic markers, in treatment monitoring, or as novel medicines for the targeted treatment of PD.

## Author contributions

JLi wrote the manuscript and worked with QZ on the conception and design. HH and JLe were responsible for literature retrieval. YS and JH contributed to the critical discussion. JW and YX polished the manuscript. All authors read and approved the submitted version.
